# Genomic and morphological analysis reveals long-term mammoth hybridization in British Columbia, Canada

**DOI:** 10.1098/rsbl.2025.0305

**Published:** 2025-09-25

**Authors:** Marianne Dehasque, Tom van der Valk, J. Camilo Chacón-Duque, Laura Termes, Petter Larsson, Hannah M. Moots, Florentine Tubbesing, Juliana Larsdotter, Gonzalo Oteo-García, Kelsey Moreland, Hans van Essen, Victoria Arbour, Grant Keddie, Michael P. Richards, David Díez-del-Molino, Peter D. Heintzman, Adrian Lister, Love Dalén

**Affiliations:** ^1^ Department of Organismal Biology, Uppsala University, Uppsala, Sweden; ^2^ Centre for Palaeogenetics, Stockholm, Sweden; ^3^ Department of Ecology and Evolutionary Biology, University of California Santa Cruz, Santa Cruz, CA, USA; ^4^ Department of Bioinformatics and Genetics, Swedish Museum of Natural History, Stockholm, Sweden; ^5^ Science for Life Laboratory, Stockholm, Sweden; ^6^ Department of Zoology, Stockholm University, Stockholm, Sweden; ^7^ Department of Archaeology, Simon Fraser University, Burnaby, Canada; ^8^ Department of Archaeology and Classical Studies, Stockholm University, Stockholm, Sweden; ^9^ Dipartimento di Biologia Ambientale, Sapienza Università di Roma, Rome, Italy; ^10^ Leiden University, Leiden, The Netherlands; ^11^ Royal British Columbia Museum, Victoria, British Columbia, Canada; ^12^ School of Earth and Ocean Sciences, University of Victoria, Victoria, British Columbia, Canada; ^13^ Department of Geological Sciences, Stockholm University, Stockholm, Sweden; ^14^ Natural History Museum, London, UK

**Keywords:** *Mammuthus*, ancient DNA, fossils, hybridization, Quaternary

## Abstract

Climate changes profoundly impact species distributions and can drastically alter dynamics between formerly isolated taxa. The evolution of mammoths within North America was characterized by repeated cycles of dispersal and putative gene flow between woolly and Columbian mammoths. However, as genome-wide studies on mammoths have predominantly focused on Siberia, the consequences of these North American range shifts remain unclear. Here, we generated genome-wide and morphological data for two Late Pleistocene mammoth molars from British Columbia, Canada (BC), and jointly analysed these with previously published data. Our genome-wide analysis (*n* = 16) revealed gene flow between woolly and Columbian mammoths that would have gone undiscovered based on morphological (*n* = 48) and mitochondrial analysis (*n* = 124) alone. Consistent with their hybrid nature, our analyses suggest that these two BC mammoths had elevated genomic diversity. Our results highlight the importance of combining data types to reconstruct past evolutionary events. These findings demonstrate how the geographical range expansion of woolly mammoths resulted in long-term hybridization with local Columbian mammoths and enhance our understanding of the genomic and morphological consequences of climate-mediated dispersal.

## 1. Introduction

The study of macroevolutionary processes in the fossil record has traditionally been based on inferences from morphological analysis [1]. The distribution and features of fossils provide insights into the ecology, evolution and dispersal of organisms, offering a glimpse into the history of life on Earth. Nevertheless, several factors, such as taphonomic biases, convergent evolution and cryptic biodiversity, may hamper inferences from morphology alone. Palaeogenomic methods and the growing availability of ancient DNA datasets now offer complementary tools to reconstruct past evolutionary processes [2–5].

The mammoth lineage (*Mammuthus* spp.) exemplifies how combining palaeontology and palaeogenomics can enhance our understanding of long-term evolutionary dynamics [6,7]. Traditionally, the classification of mammoth species is based on molar morphology, with more steppe-adapted lineages exhibiting a higher number of enamel lamellae and greater relative crown height, reflecting dietary changes over time and across geographical regions [8,9]. Recognized mammoth species that lived during the Pleistocene include the southern mammoth (*M. meridionalis*, Early Pleistocene), the steppe mammoth (*M. trogontherii*, late Early to Middle Pleistocene), the woolly mammoth (*M. primigenius*, Middle Pleistocene to Holocene) and the Columbian mammoth (*M. columbi,* Middle to Late Pleistocene).

Within Eurasia, the steppe mammoth evolved from a southern mammoth ancestor *ca* 1.8 million years ago (Myr). The steppe mammoth subsequently gave rise to the woolly mammoth around 700 thousand years ago (ka). In contrast, the evolution of North American mammoths began between 1.5 and 1.3 Myr, when mammoths with steppe-like morphology first crossed the Bering Land Bridge [9–11]. Fossil evidence traditionally suggested that Columbian mammoths were solely descended from this mammoth lineage [9]. However, ancient DNA analysis has revealed that they instead arose through a hybridization event that occurred *ca* 420 ka between woolly mammoths and a steppe mammoth population that descended from a distinct *trogontherii*-like lineage named Krestovka [6]. Notably, this resulted in all Columbian mammoth mitochondrial diversity nested within that of woolly mammoths [12–14]. Furthermore, gene flow between North American woolly mammoths and Columbian mammoths during the Late Pleistocene has also been suggested by the presence of intermediate molar morphologies [12,15,16].

Despite the evolution of mammoths within North America being a highly dynamic process characterized by repeated cycles of dispersal and putative gene flow [6,7,9,12,14], relatively little is known about the temporal continuity and extent of interbreeding between nominal woolly and Columbian mammoth species [17]. Here, we analysed two mammoth molars from British Columbia, Canada (hereafter ‘BC mammoths’), and explored their evolutionary affinities to Siberian and North American mammoths.

## 2. Material and methods

### (a) Sample collection and radiocarbon dating

Molars were sampled from the Royal British Columbia Museum (Victoria, BC) and the Okanagan Heritage Museum (Kelowna, BC), and were originally found on the Newman Peninsula of Babine Lake (RBCM.P997, S-SFU 1689; hereafter ‘*BC35.9k*’) and at Okanagan Lake (AA.71.255.1, S-SFU 1656; hereafter ‘*BC25.3k*’) (table 1, electronic supplementary materials, figure S1 and table S1). Sampling of these mammoths was performed as described in [18]. Accelerator mass spectrometry radiocarbon dating, with the inclusion of an ultrafiltration step, was performed at the Oxford Radiocarbon Accelerator Unit [19]. The radiocarbon dates were calibrated using OxCal 4.3 [20] using the IntCal20 calibration curve [21]. Dates are reported in calibrated years before present (BP).

**Table 1.** Information about two new molars from British Columbia, Canada.

sample ID	museum collection ID	lab ID	locality	median calibrated radiocarbon age (BP)	genome-wide coverage (MQ25)	genetic sex
*BC25.3k*	AA.71.255.1	bcm004	Okanagan Lake, British Columbia, Canada	25 320	8.20	XX
*BC35.9k*	RBCM.P997	bcm019	Babine Lake, British Columbia, Canada	35 876	0.37	XY

### (b) Morphological assignment

Measurements of the mammoth molars were taken according to the protocol in [9] (electronic supplementary material, S1). Comparative data are taken from [9] and comprise woolly mammoths from Alaska, Canada, and northeastern Siberia, and Columbian mammoths from sites in the contiguous USA where there is a significant sample of mammoth molars conforming to the Columbian mammoth hypodigm [15] (electronic supplementary material, S1). Two molars from the Old Crow region of Yukon Territory, Canada, that have been referred to as steppe mammoth but are similar in form to Columbian mammoths, are included for comparison.

### (c) Laboratory methods

We carried out all laboratory work at the designated ancient DNA facilities at the Centre for Palaeogenetics, Stockholm, Sweden, following standard ancient DNA guidelines to avoid and monitor for contamination. We extracted DNA from the samples following the protocol of [22]. We built double-stranded Illumina libraries [23,24], with modifications as described in [25], including uracil-removal treatment. The libraries were sequenced using paired-end chemistry on an S4 flow cell of the Illumina NovaSeq S6000 at the Science for Life Laboratory (SciLifeLab), Stockholm (electronic supplementary material, S1).

### (d) Mitogenome data processing and phylogenetic inference

The Generode pipeline (v. 2.3.1) [26] was used to pre-process raw reads by trimming adapters and merging paired-end reads. To generate a consensus mitogenome for each sample, we ran the iterative assembler MIA [27] on the pre-processed reads using the Asian elephant mitogenome (NC_005129.2) as an initial reference (electronic supplementary material, S1). Next, we used Muscle v. 3.8.31 [28] to align the new mitogenomes to the dated samples of the mitochondrial dataset from [6], after removing the most distant outgroups (*L. africana*, *L. cyclotis* and *P. antiquus*) from the original alignment. This resulted in a mitogenome alignment of the two BC mammoths, 120 other mammoths and two Asian elephants (electronic supplementary material, table S2). A section of the hypervariable control region that consists of a variable number of tandem repeats was removed from the alignment, and a Bayesian phylogenetic tree was built using BEAST v. 1.10.4 [29] (electronic supplementary material, S1). We checked for run convergence (defined as an effective sample size of >200) in Tracer v. 1.7.2 [30] and summarized the results with TreeAnnotator v1.10.

### (e) Nuclear genome data processing

We supplemented our two BC mammoths with previously published genome-wide data from eight Siberian and two North American woolly mammoths, one Columbian mammoth, two modern African savanna elephants and from a mammoth dated to around 1.4 Myr (‘Krestovka’) (electronic supplementary material, table S3) [6,7,31,32]. Raw reads were trimmed and merged using fastp v. 0.22.0 [33]. Pre-processed reads were mapped against a concatenated Asian elephant–human (EleMax1–Hg37) reference genome using bwa aln v. 0.7.17 [34] for ancient samples, and bwa mem for modern samples. BAM files were subsequently sorted and merged per sample, and duplicates and human-aligned reads were removed with samtools v. 1.17 [35].

### (f) Genetic sexing

For genetic sex determination, we used the approach presented in [36]. This approach takes into account three sources of information: reads mapping to the X chromosome (NC_064846.1), reads mapping to the Y chromosome (NC_064847.1) and the total number of reads mapping to the autosomes. Sex determination calls are made by comparing the coverage of each of the sex chromosomes in relation to the autosomal coverage (electronic supplementary material, S1).

### (g) Genome-wide heterozygosity

We used a sliding-window approach to estimate genome-wide heterozygosity following the methods of [37]. Since estimates of heterozygosity are sensitive to differences in genome-wide coverage [38,39], we restricted this analysis to a subset of high-coverage genomes. This subset consisted of the Late Pleistocene Siberian woolly mammoth dataset used in [39] and the newly generated high-coverage genome from *BC25.3k*. To mitigate biases in heterozygosity estimates, we applied additional subsampling and filtering steps (electronic supplementary material, S1) [40].

### (h) D-statistics

We tested for gene flow between mammoths using D-statistics [41]. First, we generated a pseudohaploid genome sequence for each individual in angsd v. 0.940 [42] by randomly sampling an allele (electronic supplementary material, S1). To test for potential gene flow and infer ancestry proportions, we calculated D-statistics and f4-ratios separately for the autosomes and chromosome X using AdmixTools v. 7.0.1 [43] as implemented in the R package admixr v. 0.9.1 [44]. For both analyses, we used the African savanna elephant genomes as the outgroup (electronic supplementary material, S1).

We (1) tested for excessive allele sharing between all North American mammoths [P2] and the Krestovka lineage [P3], compared to Late Pleistocene Siberian mammoths [P1], and (2) performed the same test but with the Columbian mammoth (*M. col U*) as P3 instead of Krestovka. Analyses (1) were only performed for autosomes, as there were insufficient informative sites for the X chromosome (<100). We assessed result significance (*Z* ≥ 3) using block jack-knifing in windows of 5 Mb. For the f4-ratio tests, we inferred the proportion of Columbian mammoth ancestry (1-ɑ), using samples *M. prim Wra24.0* and *M. prim Oim44.2k* as woolly mammoth donors A and B, respectively, and *M. col U* as Columbian mammoth donor C. In this test, donors B and C are considered the ancestry sources, whereas A is a sister group to B [43].

## 3. Results

### (a) Morphological assignment of the British Columbia mammoth molars

**Figure 1.**
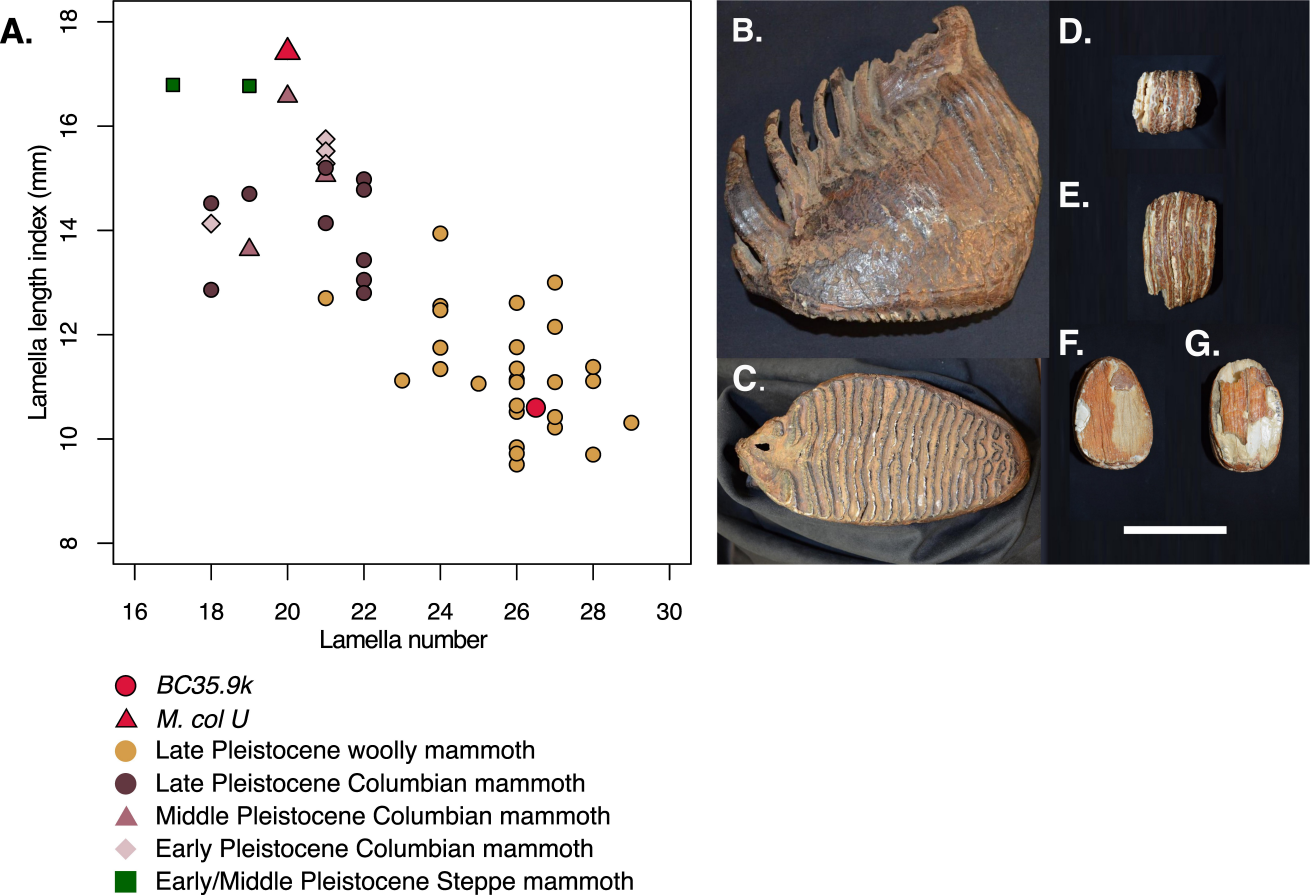
Molar morphology. (A) Bivariate plot of lamella number versus lamella length index for upper third molars of a comparative dataset of *Mammuthus* spp. (see electronic supplementary material, S2, for more details) and molar *BC35.9k* (RBCM.P997). (B) Lateral and (C) occlusal views of M3 of *BC35.9k*. (D) Occlusal, (E) lateral, (F) posterior and (G) anterior views of M3 fragment *BC25.3k* (AA.71.255.1). In (D) and (E) anterior is to the left. Scale bar (B–G) 100 mm.

Measurements of the BC mammoth molars are given in electronic supplementary material, table S4. The mammoth molar from *BC35.9k* (RBCM.P997, figure 1B,C) is an almost complete right upper last molar (M3). The lamellar number of 26−27 and lamellar length index of 10.6 are typical of woolly mammoths from Alaska, Canada, and northeastern Siberia, but well beyond the range of typical Columbian mammoths from the contiguous USA (figure 1A). *BC35.9k* can therefore be referred to *Mammuthus primigenius*. The fragment from *BC25.3k* (AA.71.255.1, electronic supplementary material, figure 1D–G) comprises only five plates that we interpret as part of the posterior half of a last molar (M3). Its average lamellar length of 14.39 is significantly higher than that of *BC35.9k* at 11.20 (electronic supplementary material, table S4), a morphology closer to Columbian mammoth than woolly mammoth upper M3s, but as the attribution of the fragment to an upper lower molar is uncertain, the specimen is identified only as *Mammuthus* sp. (see more details in electronic supplementary material, S2).

### (b) Mitochondrial clade assignment

**Figure 2.**
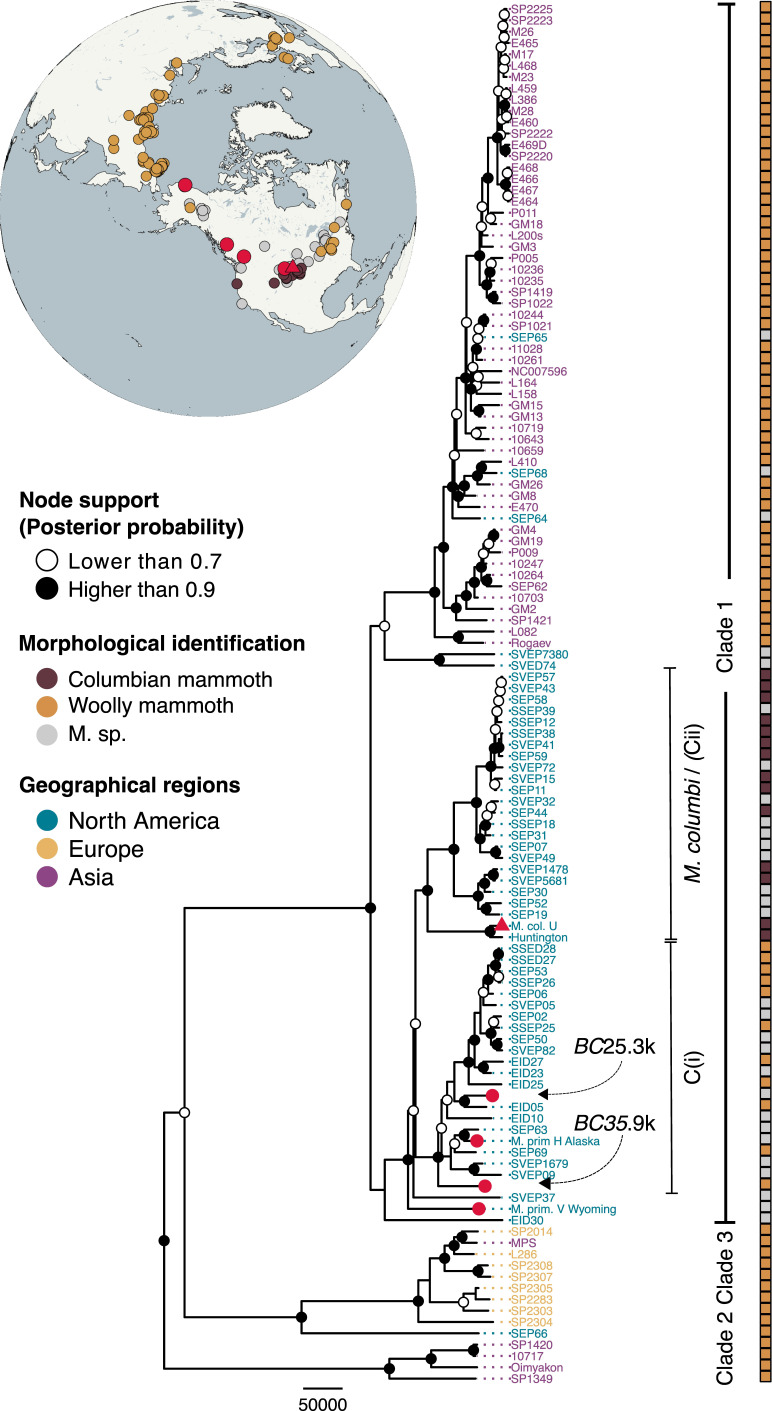
Bayesian mitochondrial phylogenetic tree with BEAST. The colour of the tip names represents the geographical region with blue = North America, yellow = Europe, and purple = Asia, with tip names representing sample IDs (see electronic supplementary material, table S2, for corresponding accession numbers). The morphological species assignments are provided as colour bars on the right with brown = Columbian mammoth, orange = woolly mammoth, and grey = unidentified. The sample localities are plotted with jitter in the globe. North American samples for which genome-wide data that is available are coloured in dark red dots (woolly mammoths) and a triangle (Columbian mammoth). The scale bar represents evolutionary rate in years. The Asian elephant outgroup samples were removed from the figure for clarity.

The assembled mitochondrial genomes had an average coverage of 68.6× for *BC35.9k* and 373.4× for *BC25.3k* [45,46]. Within our Bayesian phylogenetic framework, the three major mammoth matrilineal clades are represented and follow a phylogeographic structure (figure 2, electronic supplementary material, table S2) as previously reported [13,14]. Within Clade 1, North American mammoths in-part form two distinct clades: one morphologically assigned as woolly mammoth (haplogroup C(i)) and the other as Columbian mammoth (haplogroup C(ii)). The two Canadian specimens both fall within the North American woolly mammoth clade (haplogroup C(i)).

### (c) Genomic sexing and genome-wide heterozygosity

We generated whole-genome sequencing data [47] that yielded a genome-wide average coverage of 8.20× for *BC25.3k* and 0.37× for *BC35.9k*. Sequence-based sexing analysis showed *BC25.3k* to be a female (XX) and *BC35.9k* to be male (XY) (electronic supplementary material, table S1). We inferred genome-wide heterozygosity in *BC25.3k* and compared this value to eight Late Pleistocene Siberian woolly mammoths. The genome-wide heterozygosity in *BC35.9k* was 1.17 heterozygous sites per 1000 base pairs (bp), which is the highest measured in any woolly mammoth genome to date and 16.6% higher than the 1.0 ± 0.05 (mean ± 1σ) heterozygous sites per 1000 bp in Late Pleistocene Siberian woolly mammoths (electronic supplementary materials, figure S2 and Table S5).

### (d) D-statistics

**Figure 3.**
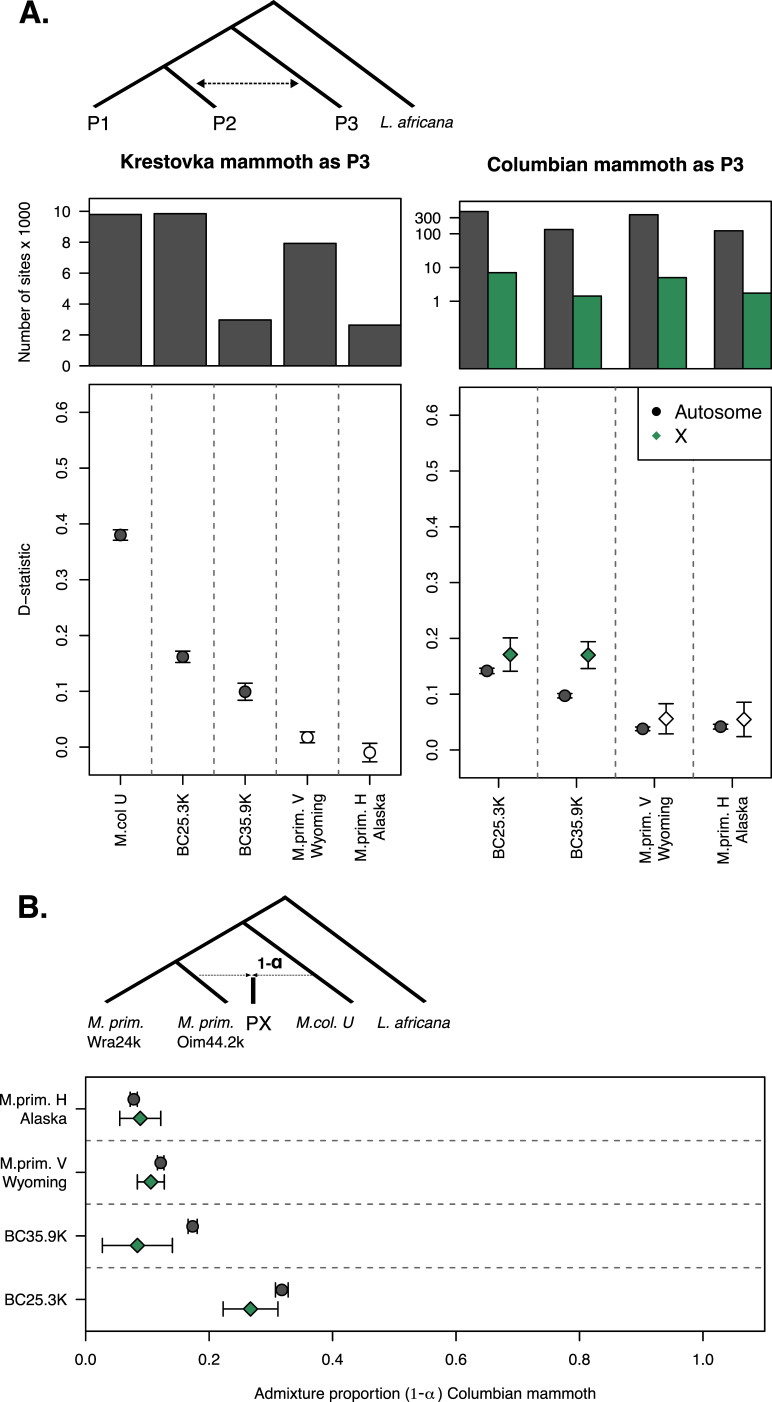
Results of D-statistics analysis and f4-ratio tests. (A) D-statistics results based on the following set-up: P1 = Siberian mammoths, P2 = *M.col* U | *BC25.3k* | *BC35.9k* | *M.prim* V Wyoming | *M.prim* H Alaska, P3 = Krestovka (left panel) or M.col U (right panel), O = *L. africana*. The bar plot of each panel represents the number of informative (ABBA + BABA) sites. The lower plots show D-statistic values (*y*-axes). Circles represent D-statistics calculated from autosomal sites, and diamonds represent D-statistics calculated from the X chromosome. Significant (*Z* ≥ 3) and non-significant (Z < 3) values are represented by closed and open symbols, respectively. Positive D-statistic values represent excessive allele sharing between P2 (*x*-axis) and P3. See also electronic supplementary materials, figures S2 and S3, for D-statistics per individual. (B) f4-ratio tests. The admixture proportions are given the following set-up: P1 = *M. prim* Wra24.0k, P2 = *M. prim* Oim44.2k, PX = *BC25.3* k | *BC35.9k* | *M.prim* V Wyoming | *M.prim* H Alaska, P3 = *M.col* U and O = *L. africana*, with 1-ɑ representing the proportion of Columbian mammoth for each individual PX given on the *y*-axis.

In the autosomal genome data, we observe excessive allele sharing between Krestovka and the Columbian and BC mammoths (*M. col U*; *BC35.9k*; *BC25.3k*), but not between Krestovka and the Wyoming and Alaskan woolly mammoths (*M. prim V*; *M. prim H*). However, all North American mammoths (*BC35.9k*; *BC25.3k*; *M. prim V*; *M. prim H*) exhibit excessive allele sharing with the Columbian mammoth (*M. col U*) (figure 3A, electronic supplementary materials, figure S3 and S4 and table S6). For the X chromosome, we detected the same excess allele sharing between Columbian mammoth and the BC mammoths, but not between Columbian mammoth and the Wyoming and Alaskan woolly mammoths.

The Columbian mammoth ancestry proportions in the North American mammoths (1-ɑ) range between 9.6% and 34.6% based on f4-ratio tests, with the highest proportions found in the two BC mammoths: *BC25.3k* (34.6 ± 0.1%) and *BC35.9k* (21.6 ± 0.9%) (figure 3B; electronic supplementary material, table S7).

## 4. Discussion

The distribution of past and present-day biodiversity in North America has been largely shaped by the periodic formation and subsequent retreat of ice sheets during glacial and interglacial cycles. During glacial cycles, the expanding Laurentide and Cordilleran ice sheets created a barrier that separated Beringia from the southern parts of the Americas, while lower sea levels exposed the Bering Land Bridge, connecting Eurasia and North America [48,49]. The appearance and disappearance of these corridors and barriers intermittently promoted population expansions and gene flow, but also isolation and local adaptation of plants and animals in refugia across geographic regions [50].

To study the biodiversity of North American mammoths in the context of these climatic fluctuations, we generated whole-genome data and morphological measurements for two mammoths from British Columbia, Canada. Genomic analysis revealed extensive gene flow between Columbian and woolly mammoths, with the youngest specimen, *BC25.3k* (AA.71.255.1), having the highest proportion of Columbian mammoth ancestry. The hybrid nature of the BC mammoths is also supported by the high genome-wide heterozygosity compared to Siberian woolly mammoths from roughly the same time period that lacked Columbian mammoth admixture. The observation that the younger BC genome (*BC25.3k*) carries approx. 60% more Columbian mammoth ancestry than the older genome (*BC35.9k*—RBCM.P997) suggests that hybridization was not a singular event, but a recurrent one leading to an increased admixture signal over time, although further samples are needed to confirm this trend.

Previous work suggested the presence of unidirectional gene flow from woolly into Columbian mammoths during the Late Pleistocene [6]. By extending the dataset with additional North American genomes, we have found evidence for multiple admixture events in both directions. To infer the direction of gene flow, the approx. 1.4 Myr specimen belonging to the distinct Krestovka lineage was critical. Since the Late Pleistocene Columbian mammoth genome derives about 40% of its ancestry from the highly divergent Krestovka lineage with the other 60% from woolly mammoths, this genome can be used as a reference to infer the direction of gene flow [6]. Excess allele sharing between woolly mammoths and Krestovka indicates gene flow from Columbian mammoths (that carry Krestovka ancestry) into the woolly mammoth lineage. Besides excess allele sharing with the Columbian mammoth, both BC mammoths share alleles with Krestovka in contrast to woolly mammoths from Wyoming and Alaska, which show only excess allele sharing with the Columbian mammoth (figure 3A). Whether this latter pattern represents gene flow from woolly to Columbian mammoths or reflects long-term ancestry sharing between North American woolly mammoths and the woolly mammoth component of the Columbian mammoth remains unresolved.

Differences in introgression levels between sex chromosomes and autosomes can indicate sex-biased gene flow. Our analyses showed equal introgression on the X chromosome and autosomes in the woolly mammoths from Wyoming and Alaska, whereas the BC mammoths had equal or less (*BC25.3k*) and less (*BC35.9k*) introgression on the X chromosome (figure 3B). Since male mammoths contribute on average one-third of the X chromosome to offspring (compared to two-thirds in females), females have a greater impact on X chromosome ancestry. This suggests that, at least for these individuals, Columbian mammoth introgression was primarily introduced through male-mediated gene flow. This is further supported by the maternally inherited mitogenomes of both BC mammoths falling within the diversity of North American woolly mammoth diversity (figure 2).

The older molar in this study, *BC35.9k*, is of woolly mammoth morphology, with no hint of Columbian morphology or intermediacy between the two (figure 1). Given the observed introgression from Columbian mammoth into this specimen, this indicates that, at least as far as molars are concerned, there was no discernible shift to Columbian-like or intermediate morphology. As molar morphology in mammals is a polygenic trait [51,52], this suggests that the degree of introgression may have been insufficient, and/or it is possible that other evolutionary forces in response to the open northern habitat and available food maintained woolly mammoth molar morphology [17]. The molar fragment from *BC25.3k,* which has an even greater degree of introgression of Columbian mammoth DNA, despite being fragmentary, may show a more Columbian-like morphology, but pending more complete material this is uncertain.

Columbian mammoths are presumed to be the result of a hybridization event around 420 ka between descendants of the Early Pleistocene Krestovka lineage and woolly mammoths in North America [17]. Nevertheless, mammoths from the contiguous USA do not exhibit a noticeable molar morphological shift throughout the Middle Pleistocene (figure 1). In the Late Pleistocene, however, they show a wide range of morphologies, mostly of typical Columbian form but some of woolly mammoth-like morphology, especially in the Midwest, where some of the molars have been classified as a different species, Jefferson’s mammoth (*Mammuthus jeffersonii*) [15,53]. Genomic analysis of this intermediate material may indicate stepwise accretion of woolly mammoth-like morphology with increasing introgression into the Columbian mammoth genome [17]. Further material from the Canadian population represented by *BC25.3k* is required to test the hypothesis of an inverse process occurring in woolly mammoth territory.

The extensive gene flow into the two BC mammoths would have gone undiscovered based on mitochondrial diversity and/or morphology alone. Yet, much of our understanding of mammoth phylogeography and evolution has been based exclusively on these two markers [9,14,16,32,53–56]. Here, we show that including genome-wide data provide a more detailed picture and lead to new insights regarding the direction and extent of hybridization. While we focus on mammoths here, the same principles likely also apply to other species. For example, the phylogeographic structure of lions, lemmings and bison have also been closely linked to past climate change and available habitat [57–59]. However, the range dynamics on a broader evolutionary and ecological scale remain unclear. While generating whole-genome data through shotgun sequencing may not be feasible for all samples, especially from more humid or warm regions, target enrichment in samples with poorly preserved DNA could provide an alternative for studying biodiversity changes through time and space [4].

Even though hybridization through climate-driven range shifts is considered a major threat to the preservation of distinct evolutionary units [60,61], the consequences on expanding and resident lineages remain poorly understood [62]. It is now evident that hybridization is an integral part of mammalian evolution and that the study of past events provides valuable insights into this process. For example, in western Europe, Iberian and brown hares have introgressed with and replaced mountain hares after the Last Glacial Maximum, revealing patterns of range expansion dynamics [63,64]. Similarly, climate-induced habitat changes promoted hybridization between polar and brown bears during the Last Glacial Maximum [65,66]. The expansion of woolly mammoths into North America provides another case study of climate-driven gene flow between formerly isolated populations. We show that woolly mammoths hybridized with Columbian mammoths, resulting in admixed populations with higher genomic diversity (electronic supplementary materials, figure S2 and table S5). While speculative, it is possible that this introgression was adaptive and even facilitated the range expansions [67]. Expanding genomic sampling across time and geography will further clarify the direction, timing and evolutionary consequences of the hybridization between these two ecologically differentiated species.

## Data Availability

Raw sequencing data and associated metadata of the two newly generated genomes have been deposited at the European Nucleotide Archive (Project PRJEB75909). The newly assembled mitogenomes and associated metadata have been deposited at GenBank (accession numbers PV752157, PV752158). The original code used to perform analyses and generate figures are available from the Zenodo repository [68]. Supplementary material is available online [69].
